# The intersection of intimate partner violence with sexual reproductive health in the Pacific: findings from a Kiribati population study

**DOI:** 10.1186/s12905-024-03484-3

**Published:** 2025-02-05

**Authors:** Anneliese Spiteri-Staines, Luz Viviana Sastre Gomez, Jess Letch, Anna Bornemisza, Kristin Diemer

**Affiliations:** 1 Department of Social Work, Melbourne School of Health Sciences, University of Melbourne, Melbourne, VIC Australia; 2 Ministry of Science and Technology, Government of Colombia, Bogota, Colombia

**Keywords:** Sexual reproductive health, Intimate partner violence, Women’s health

## Abstract

**Background:**

Women who experience intimate partner violence (IPV) are likely to experience reduced sexual and reproductive health (SRH). This paper aims to describe the prevalence of IPV and family planning use, and explore how IPV intersects with SRH among young Kiribati women; including met and unmet need for family planning, and use of contraception.

**Methods:**

Data for this paper were drawn from the Kiribati Social Development Indicator Survey [1], conducted in 2018–2019. Chi-square tests for independence were conducted, with 95% confidence intervals to identify the strength of association. Associations were considered statistically significant at *p* < .05.

**Results:**

Of the *n* = 3,106 women who had been intimately partnered or sexually active in the last year, 20% had unmet need for either spacing or limiting, the greatest unmet need being observed in women aged 15–24 years (28.8%). Half (51%) of ever-partnered Kiribati women experienced physical IPV from an ex/partner in their lifetime, one quarter experienced sexual IPV (24%) and 46% psychological IPV. Women aged 15–24 years reported higher rates of physical and/or sexual IPV over their lifetime and within the last year. Women who had experienced IPV from their partner in the last 12 months were significantly less likely to show unmet need for spacing or limiting (46.5%) than women who had not experienced any IPV (53.5%) and were more likely to be using a modern method of contraception (31%) than women who had not experienced IPV (26%). Women who experienced lifetime IPV were more also more likely to report met need for family planning.

**Conclusions:**

This study shows women in Kiribati experience elevated rates of IPV and unmet need for family planning. Inclusion of young women (including single women) and rural women, especially women living with IPV must be prioritised as an international goal if the SRH needs are to be met for all. In order to overcome the difficulties faced by young women, women in remote areas and those experiencing IPV, health-care providers would benefit from further training and information on the issues around IPV.

**Supplementary Information:**

The online version contains supplementary material available at 10.1186/s12905-024-03484-3.

## Background

The young people of Kiribati show complex needs related to poverty, geographic isolation and difficulty accessing services [2, 3]. Sexual and reproductive health (SRH) is a critical area of health and wellbeing for a community that experiences a high prevalence of intimate partner violence (IPV) against a backdrop of complex service delivery challenges in a remote small developing island state.

Sexual reproductive health (SRH) is a fundamental human right [4], however the (2018) report of the Guttmacher-*Lancet* Commission into SRH found that almost 4.3 billion people worldwide have inadequate access to SRH services over the course of their reproductive lives. This includes more than 200 million women in developing nations like Kiribati who want to avoid pregnancy but are not using modern contraception [5]. Data from population surveys demonstrate that women living in poverty are less likely to access SRH services [6, 7], with young women being the least able to access health services [8]. Access to SRH services, information and education is essential for ensuring the wellbeing, autonomy and dignity of every person.

### Geographic and demographic overview of Kiribati

Located in the central Pacific Ocean, Kiribati has a young population, with 55 per cent of the nation’s population aged under 25 years [9], making SRH issues for youth a priority. The country has a population of 119,000 people occupying just 811 square kilometres of inhabited land [10]. Many of the outer islands have limited opportunities for education and healthcare which leads to a highly mobile population with many young people moving to the main island of Tarawa. Kiribati’s main island, Tarawa, subsequently suffers from severe overcrowding [11, 12], and two thirds of the country’s population has been rated as poor or vulnerable, with population growth outpacing infrastructure and housing growth [2, 13]. The rapidly increasing population continues to put pressure on already-strained services. It is also one of the most geographically dispersed and remote countries in the world, with thirty-three atolls spread across 3.4 million square kilometres of ocean – with 3,900 km from the east to west. The provision of health services across this vast geographic area is challenging and expensive. Whilst the health status of the Kiribati population is improving over time, the country is yet to meet any of the Millennium Development Goals [14].

### Sexual and reproductive health in Kiribati

Previous SRH assessment of Kiribati revealed high potential for risks of ‘unintended pregnancies, unsafe abortions, complications during pregnancy and delivery, especially for teenage girls and women in rural areas [15]. Discussion of sexuality is often viewed as taboo in the Pacific, meaning people can find it difficult to seek information on SRH services as a result [16]. When women have less than a primary education, they are also more likely to have higher fertility and adolescent birth-rates [1].

Qualitative interviews with women in Kiribati reveal that knowledge about contraception is mixed with some women receiving information about methods from a health practitioner, others hearing information from women in the community and many demonstrating no knowledge at all [16] p71. Further, women who were interviewed and wished to avoid pregnancies were also not using contraception [16]. Studies the Pacific Islands, as well as other developing nations that are predominantly Catholic, have revealed a lack of contraceptive use due to inadequate understanding of family planning methods, mixed feelings about conceiving, and adherence to religious beliefs prohibiting contraception [17–20].

### Intimate partner violence in Kiribati

Women who experience IPV are also likely to experience significantly reduced general and reproductive health, and serious complications resulting in long-term decreased health and/or premature death [5, 21–24]. In Kiribati, studies show that rates of physical, sexual, and psychological violence against women are among the highest in the world [25]. Violence against women can include spousal violence (physical, sexual and/or psychological); sexual violence by persons other than a husband/partner, including other family members, friends, acquaintances, or strangers (i.e., non-partner sexual violence) [26, 27]. However, violence by a husband or male intimate partner (or other male family member) is the most pervasive form of violence against women globally [4, 28, 29]. Intimate partner violence is often a hidden form of violence, not reported to authorities and viewed as a private matter [30, 31]. Violence against women is more prevalent in areas where women and girls are less valued than men and contributes to the lack of empowerment of women and girls [31, 32].

The *Kiribati Family Health and Support Study (2010)* found that 68 per cent of ever-partnered Kiribati women aged 15–49 years reported having experienced at least one act of physical or sexual violence by an intimate partner across the course of their life [33–35]. This research resulted in a whole-of-government commitment to eliminate violence against women and girls, including the establishment of a dedicated Ministry for Women, Youth, Sports and Social Affairs (MWYSSA) in 2013 [36], p.5/33). The original prevalence findings have since been replicated with the *Kiribati Social Development Indicator Survey 2018–2019* (KSDIS), which found that 67 per cent of ever-partnered women reported having experienced physical or sexual violence in their lifetime, with 53 per cent reporting experiencing violence in the last twelve months [1].

### Intersection of intimate partner violence with sexual and reproductive health, and use of contraception

Use of SRH services, and specifically contraception, have been seen to be negatively impacted by the co-occurrence of IPV [37–40]. This is a global problem with significant impact on the health of women and girls, in addition to SRH [37, 41]. Existing literature reveals strong but heterogeneous relationships between IPV and the use of contraception, especially in developing countries, where both the occurrence of IPV and unmet demand for contraception are high [42–45]. Some evidence indicates that women who experience IPV are less likely to use modern contraceptive methods [46–50]. In contrast, other studies have found that women exposed to IPV are more likely to protect themselves from unwanted pregnancies [51–54]. These conflicting findings reinforce the need for further investigation of the complex relationships between IPV and contraception use [55].

Pregnancy has been particularly noted as a time where women are more at risk of IPV [23]. Traditional attitudes towards gender roles, such as the belief that men should control and dominate a relationship and household, or that women should perform domestic duties and be emotionally and physically available to men, are linked to perpetration of partner violence in pregnancy [56, 57]. On the other hand, studies have shown that women living with IPV are more likely to be using contraceptive methods to control pregnancy compared to those without [58], though partner refusal to use contraception was significantly more common among women who had ever experienced IPV [58, 59].

While national rates of IPV in Kiribati are becoming better known, we have less information about how it impacts on the lives of women, especially young women’s use of SRH services. The (2018) report of the Guttmacher-*Lancet* Commission into SRH determined that a more holistic view of SRH and adjacent issues, such as IPV and adolescent sexuality need to be addressed [5]. The recent KSDIS provides the opportunity to bringing together information about SRH needs in Kiribati alongside prevalence of IPV.

### Aims

This paper aims to describe the prevalence of IPV, describe family planning use, and explore how IPV intersects with SRH among young Kiribati women, including met and unmet need for family planning and contraceptive use.

## Methods

Data for this paper was drawn from the Kiribati Social Development Indicator Survey (KSDIS) [1], conducted in 2018–2019 as part of the Global Multiple Indicator Cluster Survey (MICS) Programme developed by UNICEF with the goal of generating internationally comparable data on women and children, covering a range of indicators including SRH, IPV and disability. Please consult the KSDIS [1] for further details on the modules within the MICS.

Research ethics to conduct this secondary analysis was obtained through the University of Melbourne research office (ethics ID 1647887.1).

### Participants

The KSDIS sample of ever-partnered women aged 18–49 years was *n* = 3,106.

### Measures

In order to explore SRH empowerment of women, we ask whether they have their contraceptive needs met, rather than merely whether they are using contraception:

### Unmet need for family planning (spacing or limiting)

The measure of **unmet need** for family planning refers to fecund women who are not using any method of contraception, but who wish to postpone the next birth and have more space between births (**spacing**) or who wish to stop childbearing altogether and limit the number of children they have (**limiting**). Unmet need is identified in MICS (from which this data is drawn) by using a set of questions eliciting current behaviours and preferences pertaining to contraceptive use, fecundity, and fertility preferences [1](pp 89–90).

### Met need for family planning (spacing or limiting)

**Met nee**d for **limiting** includes women who are using (or whose partner is using) a contraceptive method and who want no more children, are using male or female sterilisation or declare themselves as infecund.

**Met need for spacing** includes women who are using (or whose partner is using) a contraceptive method and who want to have another child or who are undecided whether to have another child. Summing the met need for spacing and limiting results in the total met need for contraception.

### Use of contraception

We report on **methods** of contraception as defined in the MICS analysis as, including **modern methods** (oral contraceptive pills, injectables, implants, intra-uterine devices (IUDs), female or male sterilisation, vaginal barrier methods (diaphragm, sponge, cervical cap, spermicidal foam, jelly, cream and sponge), emergency contraction, lactational amenorrhea method (LAM)); or **traditional methods** (periodic abstinence, withdrawal, or post-partum amenorrhea). Traditional methods are generally not accepted to be effective methods of contraception [60]. Given that traditional methods are not effective, and that the sample of Kiribati women using only traditional methods was too small for cross-tabulation amongst groups, we combined traditional methods with no method as one group for analysis.

### Analysis

In order to investigate associations between groups of women who experience violence and the types of contraceptive methods they choose, we used Chi-square tests for independence to analyse differences between groups of categorical variables, with *p-*values at the level < 0.05 reported as significant. Effect size (phi) was reported.

### Findings

#### Demographics

The sample of women who had been intimately partnered or sexually active in the last year was 3,106, with a mean age of 32.36 years (*SD* = 8.32), and a median age of 32 years, the majority of whom lived in urban areas (57.3%) (Table 1). The age of a woman was not associated with whether she lived in an urban or rural area.

**Table 1:** Percentage of partnered/sexually active women in the last year, by area and age

	Women aged 15–49 years	
**Area**	**%**	***N***
Urban	57.3	1780
Rural	42.7	1326
**Age (years)**	**%**	**N**
15–24	20.5%	638
25–34	40.3%	1252
35–49	39.2%	1216
**Total**	100%	3106

The Domestic Violence module was delivered to 2,548 women and of these women, 2,079 (81.6%) had been married or living with an intimate partner (ever-partnered). Analysis that includes IPV in this paper thus uses this sample of ever-partnered women.

### Experience of intimate partner violence

Around half (51%) of ever-partnered Kiribati women experienced physical IPV from a current or former partner in their lifetime with one quarter experiencing sexual IPV (24%) and almost half (46%) psychological IPV over their lifetime. The women who experienced IPV were significantly more likely to have a lower level of education [psychological IPV χ^2^ (2, *n* = 2079) = 5.624, *phi* = 0.06, physical or sexual χ^2^ (2, *n* = 2079) = 15.761, *p* < .01, *phi* = 0.09, physical χ^2^ (2, *n* = 2079) = 17.912, *p* < .01, *phi* = 0.09, and sexual IPV χ^2^ (2, *n* = 2079) = 11.898, *p* = .003, *phi* = 0.08)] (Appendix Table 8), live in rural areas [physical and/or sexual IPV, χ^2^(2, *n* = 2079) = 9.910, *p* < .007, *phi* = 0.069] (Appendix Table 9, with 12 months rates available here), and be aged between 15 and 24 years (χ^2^(4, *n* = 2079) = 19.106, *p* < .001, *phi* = 0.096) (Appendix Tables 10 and 12 month rates also available here).

### Family planning (use of contraception, met and unmet need)

For the analysis of need for family planning, we included women who had been either sexually active in the last year or were in a current intimate partner relationship. Need for family planning examines both the use of contraception and whether a woman’s need to manage her fertility are also being met.

### Use of contraception

Just over half of sexually active Kiribati women in a current relationship were not using any form of contraception or were using traditional methods (59.5%). Significantly more sexually active women aged 15–24 years were using no method of contraception than other age groups (74.4%) χ^2^ (4, *n* = 1188) = 21.213, *p* < .01, *phi* = 0.13 (Table 2). There were no differences between rates for urban and rural areas.

**Table 2:** Contraceptive method categories used by sexually active women/women in a current relationship

Contraceptive method	15 to 24 years	25–34 years	35–49 years	All women
No method/traditional method	74.4%	55.6%	59.8%	59.5%
Modern method	25.6%	44.4%	40.2%	40.5%
Count	82	367	739	1188

Women who experienced lifetime physical and/or sexual IPV were significantly more likely to be using a modern method of contraception (31%) than women who had not experienced lifetime IPV (26%), χ^2^ (4, *n* = 2076) = 9.498, *p* = .05, *phi* = 0.07 (Table 3). This pattern did not vary across age groups or area (Appendix Table 11).

**Table 3:** Percentage of ever-partnered women aged 18–49 who experienced physical/sexual IPV by method of contraception

	No physical and/or sexual IPV		Yes, physical and/or sexual IPV		Total	
Lifetime IPV						
	%	N	%	N	%	N
No method	68.20%	645	63.6%	713	65.8%	1366
Modern method	26.3%	272	31%	348	28.8%	597
Traditional method	5.5%	52	5.4%	61	5.4%	113
Total	100%	1870	100%	180	100%	2076
**IPV in the last 12 months**						
No method	67.3%	791	63.8%	574	65.8%	1365
Modern method	27.2%	320	30.8%	277	28.8%	597
Traditional method	5.5%	65	5.3%	48	5.4%	113
Total	100%	1176	100%	899	100%	2075

### Unmet need

17% of all women in a current relationship had unmet need for either spacing or limiting, the greatest unmet need being observed in women 15–24 years (23%), χ^2^(4, *n* = 2,852) = 35.700, *p* < .001, phi = 0.11 (Table 4). This group also had the lowest levels of using contraception for spacing or limiting (25.7%) compared to above 30% in other age groups.

**Table 4:** Unmet need of women in a current relationship, and age group in years

	15–24 years	25–34 years	35–49 years	All women
Unmet need for spacing or limiting	23%	18.8%	13.4%	17.4%
Using for spacing or limiting	25.7%	34.2%	36.8%	33.7%
No unmet need	51.4%	47%	49.7%	48.9%
Number of women	214	1,178	1,160	2852

No difference was seen in rates between urban and rural areas.

Women who had experienced any type of IPV from their partner in the last 12 months were significantly less likely to show unmet need for spacing or limiting (46.5%) than women who had not experienced any IPV (53.5%) (χ^2^ (2, *n* = 2117) = 6.69, *p* < .05, *phi* = 0.06). *Please note that totals do not add up to 100% due to missing responses.

### Met need for family planning

For this analysis, we selected women who had either been sexually active in the last year, or had a current partner, and did not want another child, or were pregnant and wish that they had delayed their pregnancy (*n* = 1,169). Forty-one per cent (40.5%) of Kiribati women reported that their needs for family planning were being met by use of modern methods of contraception, 7.4% by traditional methods, including the “no method” group.

Kiribati women who experienced IPV in their lifetime were more likely to report met need for family planning than women who have not experienced violence (Table 5) [physical IPV χ^2^ (2, *n* = 1,112) = 11.576, *p* < .003, *phi* = 0.102, sexual IPV χ^2^ (2, *n* = 1,113) = 8.131, *p* < .017, *phi* = 0.085, psychological violence, χ^2^ (2, *n* = 1,113) = 6.774, *p* < .034, *phi* = 0.078, physical or sexual IPV χ^2^ (2, *n* = 1,113) = 12.478, *p* < .002, *phi* = 0.106]. Rates of met need were not significantly different between women experiencing IPV in the last 12-months and those who had not.

**Table 5:** Women aged 18–49 who experienced physical/sexual IPV by met need for family planning

Met need for family planning by ever partnered women’s lifetime experience of IPV										
**Lifetime IPV**										
		Physical IPV		Sexual IPV		Psychological IPV		Physical or sexual IPV		
**Met need for family planning**		%	N	%	N	%	N	%	N	
	No	44.2	255	45.5	117	45	232	44.1	269	
	Yes	55.8	322	54.5	140	55	283	55.9	341	
	Total	100	577	100	257	100	515	100	610	
**IPV in the last 12 months**										
		Physical IPV		Sexual IPV		Psychological IPV		Physical or sexual IPV		
		%	N	%	N	%	N	%		N
**Met need for family planning**	No	44.7	200	44.7	101	45	198	44.8		219
	Yes	55.3	247	55.3	125	55	242	55.2		270
	Total	100	447	100	226	100	440	100		489

### IPV during pregnancy

Among Kiribati women who experienced violence, and had ever been pregnant, one in ten (10%) disclosed that violence had occurred during pregnancy. 88% of women who experienced physical violence during pregnancy reported that their current husband /partner or boyfriend perpetrated the violence. There were no significant differences between rates of rural and urban women experiencing physical IPV during pregnancy (Appendix Table 11). The sample of women was too small to report cross-tabulation of IPV during pregnancy by age, so we looked at age of marriage. Women who had married below the age of 18 were no more likely to experience IPV during pregnancy (Table 6). The mean age of childbearing in Kiribati was 31 years (2020) [61].

**Table 6:** Experience of IPV during pregnancy by age of marriage

	Age of marriage				Total	
**Violence during pregnancy**	**10–17 years**		**18–49 years**			
	%	**N**	%	**N**	%	**N**
No	87.3%	344	90.3%	1185	89.6%	1529
Yes	12.7%	50	9.7%	128	10.4%	178
Total	100%	394	100%	1313	100%	1707

*Base: ever partnered women and have been pregnant

### Contraception after birth

Women who had experienced IPV were more likely to be using current contraception after giving birth compared with women who had not experienced violence (χ^2^(2, *n* = 1921) = 6.694, *p* = .035, *phi* = 0.059) (Table 7).

**Table 7:** Experience of lifetime physical and/or sexual IPV by contraception after birth

	Lifetime physical and/or sexual IPV						
		No		Yes		Total	
		%	N	%	N	%	N
Currently using a method to avoid pregnancy	Yes	34.8	302	39.3	410	37.2	712
	No	65.2	566	60.7	634	62.8	1200
	**Total**	100	868	100	1044	100	1912

## Discussion

This paper explored the intersection of intimate partner violence (IPV) and sexual and reproductive health (SRH), in particular, issues for young women and women in remote areas. Results suggests that IPV starts early for women in Kiribati and stays with them for many years. Overall, there was little difference in rates of lifetime IPV between age groups, but greater rates of IPV in the last 12 months for women younger than 35. Rates of IPV were higher for women living in rural areas and for women who had a lower level of education, consistent with previous literature [62–65]. Women living in rural/remote areas can be at higher risk of partner violence due to the lack of options for obtaining help or leaving an unsafe relationship [64]. Further, people living in small and close-knit communities may find it more difficult to ask for assistance due to shame and reputational risk [27].

Rates of *unmet* need for family planning were high, and significantly higher for women aged under 24 years. Kiribati rates for *met* need (40.5%) were low compared to global targets of met need being 77% (high met need) [66]. Global studies in low- and middle-income countries (LMICs) have shown positive association of women’s age, and economic wealth with met need for family planning and use of modern methods of contraception [67–69]. However, the current study found no disparities between rural (where economic wealth is lower in Kiribati) and urban areas for unmet and met need for family planning.

There are studies which show higher rates of unmet need and lower rates of modern contraceptive use in rural areas of other countries [70–73]. Malarcher, Shawn and WHO (2010) identified that being in rural areas can be a barrier to access family planning services either due to a lack of services or transport to medical services where they do exist, and potentially a reluctance to attend local services in small communities where women, especially adolescents, may be worried about their privacy [74]. In Kiribati, with the rising population and remoteness of many of Kiribati’s coral atolls, the on-going challenge to provide universal access to SRH services has been acknowledged by the Ministry for Health and Medical Services [15], in particular challenges around resources and under-staffing. Despite these challenges, our study did not observe a significant difference between met/unmet family planning needs and contraceptive use in rural areas compared to urban.

A study of contraceptive utilisation amongst adolescents in Zambia showed higher use of contraceptives in rural areas compared to urban [75]. The heterogeneity of these findings may indicate that demand for and use of family planning in rural versus urban areas is more complicated than physical access to resources. Relatively few women with an unmet need for family planning in developing countries cite cost or access as reasons for not using a contraceptive method, rather tending to cite fear of side effects, breastfeeding and attitudinal factors as their main reasons [76]. Health services themselves have also been shown as limiting access to services to young people, especially in places where culture and religion were cited as the main barriers to access by unmarried adolescents [65]. The explanations underlying these reasons are not currently understood and require further exploration [72], especially given that women living in rural areas face higher risks of unintended pregnancies, complications and death compared to other women [77, 78].

The current study found that women living with IPV were more likely to be using contraceptive methods to control pregnancy and have family planning needs met compared to women without lifetime IPV. While this is consistent with some literature [58, 59, 79] there is also contrasting research that shows women who have experienced IPV endure long-term impacts on their sexual and reproductive health and sometimes have limited access to health care due to living in a controlled environment [80–83].

Studies in sub-Saharan Africa [51], Bangladesh [52], New Zealand [59], Honduras [53], and India [54] have found a positive relationship between IPV and contraception use, suggesting that women who are exposed to IPV are more likely to protect themselves from unwanted pregnancies. Some studies have also shown that women who were exposed to sexual IPV were more than twice as likely to use modern contraceptive methods than those who did not face sexual IPV [42, 43]. Similarly for other forms of IPV, women’s experience of IPV were associated with a greater use of contraception [51, 52, 84, 85].

In contrast, a growing body of research has established the linkage between IPV and contraceptive discontinuation [86]. Studies conducted in India reported results where physical violence perpetrated by the husband was associated with a lower likelihood of modern contraceptive adoption [40, 45]. A multicountry study using Demographic Health Survey (DHS) data found inconsistent results in the association between various forms of IPV and contraceptive discontinuation [87].

These conflicting findings reinforce the need for further investigation of the complex relationships between IPV and contraception use. One possible explanation for these inconsistent results is that the influence of IPV on contraceptive use is not categorical [50]. Other factors, such as cultural, societal, and relational context play moderating roles in these relationships so it can be difficult to generalise findings. For example, men who want to limit the number of children they have would be unlikely to prevent their partner from accessing contraception. Forrest et al. (2018) found that, in the context of IPV, the desire for more children among men was one of the strongest correlates of whether their wives began using contraception, even after controlling for the wives’ desire for more children [50].

Studies have observed that women experiencing controlling and dominating behaviours by their husbands are more likely to report interference in the use of contraception than those women who do not experience controlling behaviours [88]. Further research on the role of husbands’ controlling behaviours as a moderating factor in the association between IPV and the use of contraception may improve our understanding between the interplay of IPV and SRH, in particular, the use of contraception.

Women who are pregnant are at risk of experiencing IPV [89–92], though the current study did not find this to be significantly different to the risk of IPV when not pregnant. Pregnancy has been identified as a time of greater autonomy and self-awareness for women and as such pregnancy may symbolise “autonomous control over her body and her independence from her partner” [56], p. 595. Since control is a significant aspect of IPV, violent or abusive men may find pregnancy threatening and seek to re-exert control over their partners [56, 93]. Given the inconsistency of the current finding with the literature, this may be an area for further exploration.

Our finding that women who had experienced lifetime IPV were more likely to use contraception after giving birth is consistent with findings from India, where women who had experienced physical and sexual violence were more likely to have postpartum contraceptive use [94]. In situations where women have little control over sex (i.e. experience forced sex), they may be more likely to use contraception compared to women not experiencing forced sex, suggesting that they may be using contraception to gain greater control of their reproductive health in the face of loss of control over sex [42, 95]. This previous study was not limited to contraception use during the postpartum period [43], which presents another area for further exploration.

Understanding of what is driving use of different forms of contraception is challenging among a population where the majority of women are not using a method of contraception. The relationship between contraceptive use and the experience of IPV is an important intersection to explore in Kiribati given both the high rates of IPV and unmet need for family planning. Future research on this issue could offer ways to increase support for women to have safe control over their childbearing.

### Limitations

In any survey there will be respondents who opt not to disclose experiences that are asked about in the questionnaire. The more sensitive the questions are, the greater risk there is of low disclosure rates. Questions about SRH and IPV are two highly sensitive topics and we accept that any national survey will always be an underrepresentation of the true existence of the issues. Enhancing disclosure rates primarily relies on interviewer training, which can improve disclosure rates. The authors of this paper were involved in the interviewer training for the IPV module along with the Kiribati Women and Children’s Centre specialists. The training was shorter than recommended for an IPV survey but was the highest quality training possible in the time available. We accept the response rates as indicative of the issues with knowledge that they are likely underreports. Findings are limited by the cross-sectional and descriptive nature of the methodology, which prevents temporal or causal relationships from being inferred, though associative relationships were explored. These findings merit further investigation through qualitative research. Such research could delve into women’s motivations and choices regarding contraception, beyond the scope of family planning decision-making as addressed in the KSDIS questionnaire.

## Conclusion

This study contributes to the expanding body of research about the association between women’s experience with IPV and contraceptive use, and the specific SRH issues for young women. According to the data from Kiribati, women who did not use modern means of contraception were more likely to be younger but no more likely to be living remotely. Younger women were also more likely to experience IPV. However, women who experienced IPV were more likely to have met need for contraception, regardless of their age or location.

Violence in all forms can have great impact on women’s sexual and reproductive health physically, emotionally and by restricting access to health care [96]. Inclusion of young women (including single women) and rural women, especially those experiencing IPV, must be prioritised as an international goal if the SRH needs are to be met for all. Appropriate contraceptive use is important to the health of women and children as it helps to prevent pregnancies that are too early or late, limits the total number of births as well as extends the time between births for recovery (spacing). The heterogeneity of findings relating to the intersection of IPV and SRH indicates a great need to further our understanding of this complex relationship.

In order to overcome the difficulties faced by young women, women in remote areas and those experiencing IPV, health-care providers require further training and information to understand the issues around IPV. Training to manage care, provide follow up and counselling should be included in pre-service curricula, as well as access to additional counselling and legal aid. Further, governments and partner agencies can make significant contributions to the reduction of violence against women, via the promotion of SRH and IPV services that improve these women’s access.

## Electronic supplementary material

Below is the link to the electronic supplementary material.

Supplementary Material 1

## Data Availability

The questionnaire, methodology and dataset are available through the Pacific Data Hub at the following link: https://pacificdata.org/data/dataset/spc_kir_2018_mics_v01_m_v01_a_puf#:~:text=The%202018%2D19%20Kiribati%20SDIS,collect%20disaggregated%20data%20for%20the.
