# Supplementary material for: The intersection of intimate partner violence with sexual reproductive health in the Pacific: findings from a Kiribati population study
Source: BMC Womens Health. 2025 Feb 5;25:52. doi: 10.1186/s12905-024-03484-3 (PMC11796009; doi:10.1186/s12905-024-03484-3)
Supplement: Supplementary file 1 — Supplementary Material 1 [file 12905_2024_3484_MOESM1_ESM.docx]

# Appendix A:

## List of abbreviations

DV Domestic violence

IPV Intimate partner violence

SRH Sexual and reproductive health

## Appendix B

### IPV and education

Women who received an education to junior secondary level or lower experienced significantly higher rates of all forms of IPV in their lifetime compared with women educated at senior secondary level or higher [psychological IPV χ^2^ (2, *n*=2079)=5.624, *phi*=.06, physical or sexual χ^2^ (2, *n*=2079)=15.761, *p*<.01, *phi*=.09, physical χ^2^ (2, *n*=2079)=17.912, *p*<.01, *phi*=.09, and sexual IPV χ^2^ (2, *n*=2079)=11.898, *p*=.003, *phi*=.08)] (Table *8*). IPV in the last 12 months followed the same pattern with lower rates of violence among women with higher education, though only sexual IPV was statistically significant, χ^2^ (1, *n*=2079)=4.713, *p*=.03, *phi*=.05).

Table 8: Percentage of ever-partnered women by education and experience of IPV

|  | **Junior secondary or lower** | | **Senior secondary or higher** | | **Total** | |
| --- | --- | --- | --- | --- | --- | --- |
| **Lifetime IPV** | | | | | | |
|  | % | N | % | N | % | N |
| **Physical IPV** | 55.4% | 572 | 46.6% | 487 | 51.0% | 1059 |
| **Sexual IPV** | 26.6% | 275 | 20.9% | 218 | 23.7% | 493 |
| **Psychological IPV** | 48.1% | 533 | 43.5% | 583 | 45.8% | 1116 |
| **Physical or sexual IPV** | 58.1% | 600 | 50.0% | 522 | 54.0% | 1122 |
| **IPV in the last twelve months** | | | | | | |
| **Physical IPV** | 40.9% | 423 | 37.% | 394 | 39.3% | 817 |
| **Sexual IPV** | 23.3% | 241 | 19.4% | 203 | 21.4% | 444 |
| **Psychological IPV** | 39.8% | 412 | 37.2% | 389 | 38.5% | 801 |
| **Physical or sexual IPV** | 44.7% | 462 | 41.9% | 452 | 43.3% | 900 |

### Area and experience of IPV

In Kiribati, differences between rural and urban areas were significant for lifetime IPV, with rates being higher in rural areas for physical IPV, χ^2^ (2, *n*=2079) = 7.915, *p*<.019, *phi*= 0.062, physical and/or sexual IPV, χ^2^(2, *n*=2079) = 9.910, *p*<.007, *phi*= 0.069, and sexual IPV, χ^2^ (2, *n*=2079) = 7.915, *p*<.01 Table 9: Percentage 9, *phi*= 0.062 (Table 9).Variation for IPV in the last 12 months was not significant by area.

Table 9: Percentage of ever-partnered women by area and experience of IPV.

|  | **Urban** | | **Rural** | | **Total** | |
| --- | --- | --- | --- | --- | --- | --- |
| **Lifetime IPV** | | | | | | |
|  | % | N | % | N | % | N |
| **Physical IPV** | 49.2% | 502 | 53.1% | 557 | 51.2% | 1059 |
| **Sexual IPV** | 22.5% | 232 | 24.9% | 261 | 23.7% | 493 |
| **Psychological IPV** | 45.8% | 472 | 45.8% | 481 | 45.8% | 953 |
| **Physical or sexual IPV** | 51.7% | 533 | 56.1% | 589 | 54.0% | 1122 |
| **IPV in the last twelve months** | | | | | | |
| **Physical IPV** | 38.6% | 397 | 39.9% | 419 | 39.3% | 816 |
| **Sexual IPV** | 20.2% | 208 | 22.5% | 236 | 21.4% | 444 |
| **Psychological IPV** | 38.7% | 399 | 38.3% | 402 | 38.5% | 801 |
| **Physical or sexual IPV** | 42.5% | 437 | 44.1% | 463 | 43.3% | 900 |

Younger women (15-24 years) reported higher rates of physical, χ^2^(4, *n*=2079)= 16.538, *p*<.002, *phi*= 0.089), sexual, χ^2^(4, *n*=2079)= 10.448, *p*<.034, *phi*= 0.031, and physical or sexual IPV, χ^2^(4, *n*=2079)= 19.106, *p*<.001, *phi*= 0.096, in their lifetime than women aged 25 to 49 years (Table 10). Similar results were seen for IPV during the last twelve months, including physical, χ^2^ (2, *n*=2079)= 47.630, *p*<.001, *phi*= 0.151, sexual, χ^2^ (2, *n*=2079)= 13.502, *p*<.001, *phi*= 0.081, physical or sexual, χ^2^ (2, *n*=2079)= 45.300, *p*<.001, *phi*= 0.14, and also psychological IPV, χ^2^ (2, *n*=2079)= 8.139, *p*<.007, *phi*= 0.063.

Table 10: Percentage of ever-partnered women by age and experience of IPV

|  | **15-24 years** | | **25-34 years** | | **35-49 years** | | **Total** | |
| --- | --- | --- | --- | --- | --- | --- | --- | --- |
| **Lifetime IPV** | | | | | | | | |
|  | % | N | % | N | % | N | % | N |
| **Physical IPV** | 57.9% | 184 | 53.3% | 466 | 46.1% | 409 | 50.9% | 1059 |
| **Sexual IPV** | 28.4% | 90 | 25.3% | 221 | 20.5% | 182 | 23.7% | 493 |
| **Psychological IPV** | 47.6% | 151 | 47.3% | 414 | 43.7% | 388 | 45.8% | 953 |
| **Physical or sexual IPV** | 61.8% | 196 | 56.3% | 492 | 48.9% | 434 | 54.0% | 1122 |
| **IPV in the last twelve months** | | | | | | | | |
| **Physical IPV** | 50.6% | 161 | 43.4% | 379 | 31.2% | 277 | 39.3% | 817 |
| **Sexual IPV** | 26.2% | 83 | 23.3% | 204 | 17.7% | 157 | 21.4% | 444 |
| **Psychological IPV** | 43.2% | 137 | 40.2% | 351 | 35.2% | 312 | 38.5% | 800 |
| **Physical or sexual IPV** | 54.9% | 174 | 47.2% | 413 | 35.4% | 314 | 43.3% | 901 |

Table 11: Percentage *of ever-partnered women aged 18-49 who experienced physical/sexual IPV by method of contraception and age group*

| **Physical and/or sexual IPV** | | | | | | | |
| --- | --- | --- | --- | --- | --- | --- | --- |
|  | | **No** | | **Yes** | | **Total** | |
|  | | % | N | % | N | % | N |
| **15- 24 years old** | No method | 82.5% | 99 | 70.8% | 138 | 75.2% | 237 |
|  | Modern method | 16.7% | 20 | 26.7% | 52 | 22.9% | 72 |
|  | Traditional method | 0.8% | 1 | 2.6% | 5 | 1.9 | 6 |
|  | **Total** | 100% | 120 | 100% | 195 | 100% | 315 |
| **25-34 years old** | No method | 65.7% | 249 | 62.7% | 309 | 64.0% | 558 |
|  | Modern method | 27.4% | 104 | 32.0% | 158 | 30.0% | 262 |
|  | Traditional method | 6.9% | 26 | 5.3% | 26 | 6.0% | 52 |
|  | **Total** | 100% | 379 | 100% | 493 | 100% | 872 |
| **35- 49 years old** | No method | 66.6% | 297 | 61.4% | 266 | 64.1% | 563 |
|  | Modern method | 27.8% | 124 | 31.9% | 138 | 29.8% | 262 |
|  | Traditional method | 5.6% | 25 | 6.7% | 29 | 6.10% | 54 |
|  | **Total** | 100% | 446 | 100% | 433 | 100% | 879 |
| **Total** | **No method** | 68.3% | 645 | 63.6% | 713 | 65.7% | 1358 |
|  | **Modern method** | 26.2% | 248 | 31.0% | 348 | 28.8% | 596 |
|  | **Traditional method** | 5.5% | 52 | 5.4% | 60 | 5.4% | 112 |
|  | **Total** | 100% | 945 | 100% | 1121 | 100% | 2066 |

Table 12: *Percentage of ever-partnered women aged 18-49 who experienced physical/sexual IPV by method of contraception and area*

| **Physical and/or sexual IPV** | | | | | | | |
| --- | --- | --- | --- | --- | --- | --- | --- |
|  | | **No** | | **Yes** | | **Total** | |
|  | | % | N | % | N | % | N |
| **Urban** | No method | 70.2% | 341 | 64.8% | 344 | 67.4 | 685 |
|  | Modern method | 25.7% | 125 | 30.7% | 163 | 28.3% | 288 |
|  | Traditional method | 4.1% | 20 | 4.5% | 24 | 4.3% | 44 |
|  | **Total** | 100% | 486 | 100% | 531 | 100% | 1017 |
| **Rural** | No method | 66.1% | 304 | 62.5% | 369 | 64.1% | 673 |
|  | Modern method | 27% | 124 | 31.4% | 185 | 29.4% | 309 |
|  | Traditional method | 7.0% | 32 | 6.1% | 36 | 6.5% | 68 |
|  | **Total** | 100% | 460 | 100% | 590 | 100% | 1050 |
| **Total** | **No method** | 68.2% | 645 | 63.6% | 713 | 65.7% | 1358 |
|  | **Modern method** | 26.3% | 249 | 31.0% | 348 | 28.9% | 597 |
|  | **Traditional method** | 5.5% | 52 | 5.4% | 60 | 5.4% | 112 |
|  | **Total** | 100% | 946 | 100% | 1121 | 100% | 2067 |
